# Comparison of hybrid-and mono-pathotype *Escherichia coli* isolates from South Korea based on whole genome analysis and cytotoxicity assay

**DOI:** 10.1186/s12929-026-01243-0

**Published:** 2026-04-13

**Authors:** Jong Hyun Shin, Min Seok Kim, Ji Young Choi, Sunju Kim, Kun Taek Park, Mi-Ran Seo, Seung-Hyun Jung, Yeun-Jun Chung, Hyeri Seok, Hae Suk Cheong, Ki-Tae Kwon, Bo Kyeung Jung, Cheol-In Kang, Doo-Ryeon Chung, Dongwoo Shin, Kwan Soo Ko

**Affiliations:** 1 Department of Microbiology, Sungkyunkwan University School of Medicine, Suwon, 16419 Republic of Korea; 2 Antimicrobial Resistance Laboratory, Institut Pasteur Korea, Seongnam, 13488 Republic of Korea; 3 Department of Biotechnology, Inje University, Gimhae, 50834 Republic of Korea; 4 ConnectaGen, Hanam, 12918 Republic of Korea; 5 Department of Biochemistry, College of Medicine, The Catholic University of Korea, Seoul, 06591 Republic of Korea; 6 Department of Microbiology, College of Medicine, The Catholic University of Korea, Seoul, 06591 Republic of Korea; 7 Division of Infectious Diseases, Department of Medicine, Korea University Ansan Hospital, Korean University College of Medicine, Ansan, 15355 Republic of Korea; 8 Division of Infectious Disease, Department of Internal Medicine, Kangbuk Samsung Hospital, Sungkyunkwan University School of Medicine, Seoul, 03181 Korea; 9 Department of Internal Medicine, School of Medicine, Kyungpook National University, Daegu, Republic of Korea; 10 Department of Laboratory Medicine, Dankook University College of Medicine, Cheonan, 31116 Republic of Korea; 11 Division of Infectious Diseases, Samsung Medical Center, Sungkyunkwan University School of Medicine, Seoul, Korea

**Keywords:** Pathogenic *Escherichia coli*, Hybrid-pathotype, Whole genome sequencing, Virulence, Cytotoxicity

## Abstract

**Background:**

Among intestinal pathogenic *Escherichia coli*, hybrid pathotypes carrying virulence determinants of multiple classical pathotypes have recently been identified.

**Methods:**

A total of 292 *E. coli* isolates were collected from human, livestock, and environmental sources in South Korea, and their whole genome sequences were determined. They were classified into pathotypes based on virulence gene profiles. We constructed a phylogenetic tree based on cgSNPs. Synteny analysis on the LEE pathogenicity island was performed, and enriched accessory gene repertoires in particular hybrid-pathotypes were identified. Bacterial virulence was measured by lactate dehydrogenase (LDH) cytotoxicity assays. Genetic factors associated with increased cytotoxicity in the LDH assay were identified using SPLASH.

**Results:**

Among 192 intestinal pathogenic *E. coli* isolates, 63 hybrid pathotype isolates (38 STEC/EPEC, 23 STEC/ETEC, and 2 STEC/EAEC) were identified. Two main groups of STEC/EPEC hybrid-pathotype isolates and one major STEC/ETEC group were identified. Both groups of STEC/EPEC hybrid isolates were closely associated with EPEC mono-pathotype isolates, and most STEC/ETEC hybrid groups clustered with STEC mono-pathotype isolates. Synteny analysis on the LEE pathogenicity island also showed that some STEC/EPEC hybrids might originate from EPEC genomic backbone. Based on the LDH assay, cytotoxicity was higher in STEC/ETEC, but lower in STEC/EPEC. Five gene modules (adhesion-pili, plasmid conjugation, flagella-motility, iron uptake, and T3SS) were identified to be genetic factors correlated with increased cytotoxicity in STEC/ETEC.

**Conclusion:**

High prevalence of hybrid-pathotype *E coli* isolates was identified. They might emerge repeatedly by independent incorporation of additional virulence factors, and genomic backbones and cytotoxicity differed according to the specific hybrid pathotype.

**Supplementary Information:**

The online version contains supplementary material available at 10.1186/s12929-026-01243-0.

## Introduction

*Escherichia coli* is a facultative anaerobe that primarily inhabits the gastrointestinal tracts of humans and animals. Many *E. coli* strains support the commensal balance of the gut microbiota, but some pathogenic strains have developed to cause serious extraintestinal and enteric illnesses [1, 2]. The intestinal pathogenic *E. coli* strains have been categorized into six well-described pathotypes, enteropathogenic *E. coli* (EPEC), enterotoxigenic *E. coli* (ETEC), enterohaemorragic *E. coli* (EHEC), enteroinvasive *E. coli* (EIEC), enteroaggregative *E. coli* (EAEC), and diffusely adherent *E. coli* (DAEC) [1]. In addition to them, further pathogenic *E. coli* strains have been described, such as Shiga toxin-producing *E. coli* (STEC) and extraintestinal pathogenic *E. coli* (ExPEC) [3]. Recently, EFSA BIOHAZ Panel recommended that the EHEC terminology as a subset of STEC is obsolete and should be replaced with STEC [4]. Pathogenicity islands, phages, and plasmids encode distinct virulence factors that mediate the pathogenic mechanisms of these pathotypes [5].

EPEC, including *eae* and/or *bfp* as virulence markers, primarily causes infantile diarrhea, lasting more than two weeks, and ETEC with LT and/or ST is known to the main cause of traveler’s diarrhea usually with no fever. Symptom of Shiga toxin (*stx*)-producing pathotypes, EHEC and STEC, starts as watery diarrhea but often progresses to severe bloody diarrhea, that is, hemorrhagic colitis. Hemolytic uremic syndrome (HUS), causing kidney failure, can result from them. While EAEC infections are characterized by persistent watery diarrhea with low-grade fever, EIEC causes watery diarrhea that may become bloody with mucus, accompanied by fever, chills, and severe abdominal cramps. EAEC represents *aggR*/*aatA*/*aaiC* and the stacked-brick adherence pattern, and the virulence marker of EIEC is *ipaH* in EIEC [6, 7]. Many of these virulence determinants are carried on mobile genetic elements, including plasmids, bacteriophages, and pathogenicity islands, which facilitate horizontal gene transfer across *E. coli* pathotypes and contribute to the emergence of hybrid strains [8].

Recently, hybrid-pathotypes that harbor virulence factors of two or more pathotypes have been identified, complicating our knowledge on *E. coli* pathogenesis [6]. The hybrids frequently exhibit increased virulence and adaptability as a result of acquiring genetic components from several pathotypes [9, 10]. For instance, German outbreak in 2011 was caused by the hybrid EHEC O104:H4 strain, which combined aggregative adherence factors from EAEC with Shiga toxin genes from STEC [11].

In this study, we focused on two hybrid-pathotypes, ETEC/STEC and EPEC/STEC. We examined the whole-genome sequences of intestinal pathogenic *E. coli* isolates from South Korea. Based on the whole genome sequences, we investigated the evolutionary positions of hybrid pathogenic isolates. In addition, we evaluated their virulence potentials using lactate dehydrogenase (LDH) assays. We found that hybrid pathogenic isolates did not emerge through a single event and they exhibited high virulence potential compared to the isolates with mono-pathotype.

## Materials and methods

### Pathogenic *E. coli* isolates

A total of 292 *E. coli* isolates were collected from humans and environments associated with livestock in South Korea between 2000 and 2024. Among them, 50 isolates from humans were obtained from several hospitals including Kangbuk Samsung Hospital (Seoul), Samsung Medical Center (Seoul), Korea University Ansan Hospital (Ansan), Kyungpook National University Hospital (Daegu), Dangook University Hospital (Cheonan), and Samsung Changwon Hospital (Changwon). The other 242 isolates were obtained from chicken (77 isolates), pigs (137 isolates), and environments around slaughterhouses and pig farms (28 isolates) (Table 1).

**Table 1:** Total distribution of pathotypes based on specific virulence genes and sources

Pathotype	Target gene	Human	Animal		Environment	Number of isolates (% among pathogenic 193 isolates)
			Chicken	Pig		
Non-pathotype		3	1	74	21	99
Mono-pathotype						
STEC	*stx*	6		18		24 (12.4)
EPEC	*eae, bfpA*		76	19	7	102 (52.8)
ETEC	LT or ST	1		3		4 (2.1)
Hybrid-pathotype						
STEC/EPE C	*stx, eae, bfp*	38				38 (19.7)
STEC/ETEC	*stx,* LT or ST			23		23 (11.9)
STEC/EAEC	*aggR, stx*	2				2 (1.0)
Total		50	77	137	28	292

### In silico analysis: de novo assembly, pathotyping, serotyping, and multilocus sequence typing

Genomic DNA was isolated from 292 *E. coli* isolates using the iDetect gDNA Prep Kit for Microbes (ConnectaGen Inc., Hanam, South Korea). Library quality and quantity were assessed using a TapeStation 4200 system (Agilent, CA, USA) with the KAPA Library Quantification Kit (Roche, Switzerland). Sequencing was carried out on the Illumina NovaSeq 6000 platform with 150 bp paired-end reads, in accordance with the manufacturer's protocol. Whole genome sequencing was performed with the ThermoFisher Scientific Ion S5 system, and the raw paired-end reads were analyzed using bioinformatics tools on a Linux platform. Initially, quality control of the raw reads was conducted using FastQC (http://www.bioinformatics.babraham.ac.uk/projects/fastqc). Adapter sequences and low-quality bases were removed using Trimmomatic v0.39 [12]. Subsequently, paired-end reads were assembled with SPAdes v3.15.5 [13].

Pathotype and serotype classification was performed using the assembled genomes. The classification criteria included five mono-pathotypes and hybrid-pathotypes as follows: EAEC (*aggR*), STEC (*stx*), EPEC (*eae*, *bfp*), ETEC (LT or ST), STEC/EPEC (*stx, eae, bfp*), and STEC/ETEC (*stx*, LT or ST). EPEC has been classified in two categories; while isolates with both *eae* and *bfpA* was defined as typical EPEC (tEPEC), the isolates with only *eae* was classified into atypical EPEC (aEPEC). *stx* gene subtypes were determined by BLASTn analysis [14]. As recommended by ESFA BIOHASZ Panel, we used a term STEC consistently instead of EHEC [4].

The classifications were further verified using ABRicate v1.0.1 (https://github.com/tseemann/abricate) and cross-validated with publicly available web tools hosted by the Center for Genomic Epidemiology (CGE) (https://www.genomicepidemiology.org/), specifically VirulenceFinder-2.0 Server (software version 2.0.5, database version 2022-12-02). Both groups of STEC/EPEC isolates from humans were clustered with EPEC mono-pathotype isolates from porcine sources using ECTyper v2.0.0, and results were cross-validated using SerotypeFinder v2.0.1 (database version 1.0.0, dated 2022-05-16; Center for Genomic Epidemiology, DTU) [15]. Only isolates with concordant serotype calls from both tools were included in the final analysis. Based on the whole genome sequences, multilocus sequence typing (MLST) was performed and sequence types (STs) were identified [16].

### Core gene SNPs and phylogenetic analysis

We used Snippy v4.6.0 (https://github.com/tseemann/snippy) and Snippy-core to extract and align core gene single nucleotide polymorphisms (cgSNPs) from all *E. coli* isolates, utilizing raw FASTQ files for the analysis. The reference genome used for this process was *E. coli* O157:H7 strain F8092B, a pathogenic *E. coli* reference, with the accession number CP038355.

A maximum-likelihood (ML) phylogenetic tree was constructed using IQ-tree v2.0.3 based on the cgSNPs [17]. The resulting phylogenetic tree was visualized using FigTree v1.4.4 (http://tree.bio.ed.ac.uk/software/figtree/).

### Synteny analysis and virulence profiling

Open reading frames were predicted from each assembly with Prodigal to generate per-sample amino-acid FASTA files [18]. All downstream protein-based screens used these predicted proteins. We detected secretion systems with MacSyFinder v2 using the TXSScan model collection [19]. We screened assemblies against the VFDB database using ABRicate with thresholds of ≥ 90% identity and ≥ 60% query coverage. From the VFDB results, we summarized several key virulence panels. The Locus of Enterocyte Effacement (LEE) panel was extensive, comprising core components (*escC/V/N*), a translocon (*espA/B/D*), chaperones (e.g., *cesD*, *cesT*), regulators (e.g., *ler*, *grlA*), numerous effectors (e.g., *tir*, *map*, *espF*), and intimin (*eae*) [20, 21]. Additionally, we identified pEHEC plasmid components (*ehxA*, *espP*, *katP*, *toxB*, and *stcE*) and the Long Polar Fimbriae gene (*lpfA*) [1, 22, 23]. Finally, an iron acquisition and heme uptake modules were defined by aggregating gene-sets for enterobactin, aerobactin, yersiniabactin (HPI), and other related transport systems (ferrichrome, ferric-citrate, and heme uptake) [24]. Clinker was used to visualize synteny analysis [25]. We also constructed a minimum spanning tree for the LEE pathogenicity island using SNP distances calculated by the snp-dists tool, followed by network visualization with the igraph package in R version 4.2.3. Integration sites of the LEE pathogenicity island were identified by performing BLASTn searches using sequences from both flanking ends of the LEE-containing contig [26].

We implemented a tiered matching strategy to ensure sufficient coverage. The primary acceptance criteria were set at a percent identity of ≥ 90% and a query coverage of ≥ 90%. In cases where these stringent parameters yielded insufficient data, the thresholds were lowered to a percent identity of ≥ 85% and a query coverage of ≥ 80%. A final, more permissive step accepted matches based on identity alone (≥ 85%). In addition to these panels, we identified gene groups related to other functions. These included genes for flagella/motility (*fliY, fliZ, flgN*, and *fliD*), plasmid conjugation (*traJ* and *traT*), adhesion pili (*ycbT*, and *ycbS*), a T3SS effector (*espX5 and ecpC*), and iron uptake (*fepA*).

### Lactate dehydrogenase assay

Virulence of pathogenic *E. coli* isolates was measured by lactate dehydrogenase (LDH) assay [27]. J774A.1 cells were prepared at a concentration of 1 × 10^5^ cells/mL and seeded at 100 µL per well in a 96-well plate, followed by incubation at 37 °C for 20 h. A single colony of each *E. coli* isolate, obtained from an overnight culture on an LB agar plate, was inoculated into 3 mL of Luria–Bertani (LB) broth and cultured at 37 °C with shaking for 24 h. On the next day, the overnight bacterial cultures were diluted to an OD of 0.05 at 600 nm. The supernatant from the 96-well plate was removed, and the diluted bacterial suspension was added to each well in duplicate at 100 µL per well. The LDH cytotoxicity assay kit (Abcam, Cambridge, UK) was used to prepare the necessary controls. After centrifuging the plate at 1,000 rpm for 5 min, the plate was incubated at 37 °C for 1 h. After incubation, 10 µL of the supernatant from each well was transferred to a new 96-well plate. To each well of the new plate, 100 µL of substrate mix or water-soluble tetrazolium salts (WSTs) substrate mix was added. The plate was covered with foil and incubated at 37 °C for 30 min. Finally, the absorbance was measured at 450 nm using a plate reader.

### SPLASH analysis

To identify sample-associated sequence variation, SPLASH2 was applied to raw sequencing reads to detect constant “anchor” k-mers and their linked, sample-specific target sequences [28]. The raw SPLASH outputs were converted into an anchor-target count table, and low-abundance anchor-target entries were filtered to improve signal quality. To calculate target fractions, the target counts were normalized to the total counts of all targets linked to the same anchor for each sample. For each anchor, constant targets observed in all samples were excluded.

Among the remaining targets for each anchor, the target observed in the largest number of samples was defined as the most prevalent target (type A). For each sample, a dominant target was assigned only when the major target fraction exceeded 0.5. Otherwise, the sample was labeled as mixed or ambiguous and excluded from target-type comparisons. Sequence variations identified within specific virulence loci were mapped and grouped. Strains carrying the most frequently observed sequence variant for a given gene were designated as the ‘most-prevalent-target’ group (representing the major allele). Strains harboring alternative sequence variations at the same locus were classified into the ‘other-targets’ group (representing minor alleles or non-dominant sequence types). Subsequently, the LDH cytotoxicity of these groups were evaluated using nonparametric tests to identify any potential group differences. The LDH cytotoxicity distributions were then compared between the two groups using a two-sided Mann–Whitney U test. Multiple testing correction across anchors was performed using the Benjamini–Hochberg false discovery rate (FDR) procedure.

For annotation, BLASTn searches were performed against the *E. coli* virulence factor database from Abricate (https://github.com/phac-nml/ecoli_vf) using the anchor–target sequence representation. A tiered matching strategy was applied: the primary threshold required ≥ 90% identity and ≥ 90% query coverage; if no qualifying hit was identified, relaxed thresholds of ≥ 85% identity and ≥ 80% query coverage were accepted. Anchors meeting these criteria were assigned to virulence factors to generate an anchor-to-gene dictionary. For each annotated gene, LDH cytotoxicity was summarized and visualized as boxplots comparing the most prevalent target group with the other targets group.

### Statistical analysis

Statistical analyses were performed using Python 3.9, utilizing libraries including SciPy [29] and scikit-posthocs [30]. Comparisons of gene presence/absence were performed using Fisher’s exact test, with multiple testing controlled by the Benjamini–Hochberg false discovery rate (FDR). Comparisons of LDH values between carriers and non-carriers were conducted primarily with the Mann–Whitney U test, supplemented by Welch’s *t*-test when appropriate. Cytotoxicity values among pathotypes were compared using the Kruskal–Wallis test with Dunn's multiple comparisons post-hoc test, as data were non-normally distributed with unequal variances across groups. Statistical analyses were performed using GraphPad Prism v9.0.0. Statistical significance was indicated as follows: *p < 0.05; **p < 0.01; ***p < 0.001; ****p < 0.0001.

## Results

### Distribution of pathotypes

In this study of 292 *E. coli* isolates from South Korea, three diarrheagenic pathotypes (STEC, EPEC, and ETEC) were identified in 193 intestinal pathogenic isolates, whereas pathotype defining markers were not detected in 99 isolates, which were designated “non-pathotype” (Table 1). Including hybrid-pathotype, the most common pathotype was EPEC. As all EPEC isolates included in this study possessed only *eae*, they were classified into aEPEC. A total of 140 isolates were classified as aEPEC (47.9%, 72.5% among pathogenic *E. coli* isolates), followed by STEC with *stx* (87 isolates). Two types of *stx*, *stx1* and *stx2*, were identified; *stx1* and *stx2* were found in 38 and 75 STEC isolates, respectively. While twelve STEC isolates possessed only *stx1* gene, 49 isolates harbored only *stx2* gene. 26 STEC isolates harbored the two subtypes of *stx*. Twenty-seven ETEC isolates, harboring LT or ST, were identified; ST was identified in all ETEC isolates, but LT was found in only two isolates. Two isolates with *aggR*, which is designated EAEC, were identified.

Among the 193 pathogenic *E. coli* isolates, hybrid-pathotypes harboring virulence factors of two pathotypes were identified in 63 isolates (32.6%) (Table 1). The most frequently identified hybrid-pathotype was STEC/EPEC (38 isolates, 19.7%), and the STEC/ETEC hybrid pathotype was identified in 23 isolates (11.9%), in which only *stx2* was identified. STEC/EAEC hybrid-pathotype was found in two isolates.

Among the 87 STEC isolates, 63 isolates (72.4%) carried virulence factors of other pathotypes, indicating hybrid-pathotypes. Thus, 24 isolates represented STEC mono-pathotype, and only four isolates exhibited ETEC mono-pathotype. Three out of four ETEC mono-pathotype isolates contained both heat-labile (LT) and heat-stable (ST) enterotoxins, but only ST was identified in all isolates of STEC/ETEC hybrid-pathotype.

While all STEC/EPEC hybrid-pathotype isolates were obtained from humans, the STEC/ETEC hybrid-pathotype was identified only in porcine isolates. The EPEC mono-pathotype was not identified in human isolates, whereas all pathogenic *E. coli* isolates from chickens were classified as EPEC (Table 1).

### Serotypes and STs

Very diverse serotypes were identified in the 193 intestinal pathogenic *E. coli* isolates. Notably, mono-pathotype isolates exhibited greater serotype diversity than hybrid-pathotype isolates.

EPEC mono-pathotype showed the most diverse serotypes. Among the 102 EPEC mono-pathotype isolates, 19 O-antigen types were identified, whereas 52 isolates lacked identifiable O-antigen (Supplementary Table S1). For H-antigen, 15 H-antigen types were identified, and 7 isolates had no detectable. The predominant serotype among EPEC mono-pathotype isolates was –:H21, accounting for 32 of 102 isolates (31.4%). Among the 24 STEC mono-pathotype isolates, eight O-antigen types were identified and four STEC isolates (16.7%) lacked an O-antigen (Supplementary Table S2). Nine H-antigen types were detected. The predominant serotype among STEC mono-pathotype isolates was O9:H9 (5 of 24 isolates, 20.8%). Among the ETEC mono-pathotype isolates, four O-antigen types were detected, and no isolates lacked an O-antigen (Supplementary Table S3). Four H-antigen types were also found. Distribution of non-pathotype *E. coli* across different O-antigens and H-antigens was shown in Supplementary Table S4.

In STEC/EPEC hybrid isolates, only five serotypes were identified, with O157:H7 as the predominant serotype (22 out of 38 isolates, 57.9%) (Supplementary Table S5). The whole genome sequences of STEC/EPEC O157:H7 isolates in this study showed ≥ 99.87% nucleotide identity, and they showed the nucleotide identities of 97.3% to 99.45% with the O157:H7 reference strain Sakai [31]. Similarly, eight serotypes were detected among STEC/ETEC hybrid isolates (Supplementary Table S6). O100:H30 appeared in eight STEC/ETEC isolates. Two STEC/EAEC hybrid isolates shared O104:H4 serotype. While the two STEC/EAEC O104:H4 isolates included in this study showed 99.98% nucleotide identity, they exhibited nucleotide identifies of 99.76% and 99.80% with the German outbreak O104:H4 strain LB226692, respectively.

Among the 292 *E. coli* isolates, 65 STs were identified based on the whole genome sequences (Supplementary Table S7). There was no complete exclusive correlation between ST and pathotype. In EPEC isolates, ST752 was predominantly found (66.7%, 68/102), followed by ST20 (seven isolates) and ST48 (five isolates). While ST10 was the most frequently identified among the STEC isolates (25.0%, 6/24), ST11 was the most abundant among the STEC/EPEC hybrid isolates (57.9%, 22/38). The ST11 was also identified in three STEC mono-pathotype isolates, but was not found in EPEC mono-pathotype isolates. In STEC/ETEC, ST993 was predominantly found (34.8%, 8/23), followed by ST3630 (six isolates).

### Phylogenetic analysis

**Fig. 1:**
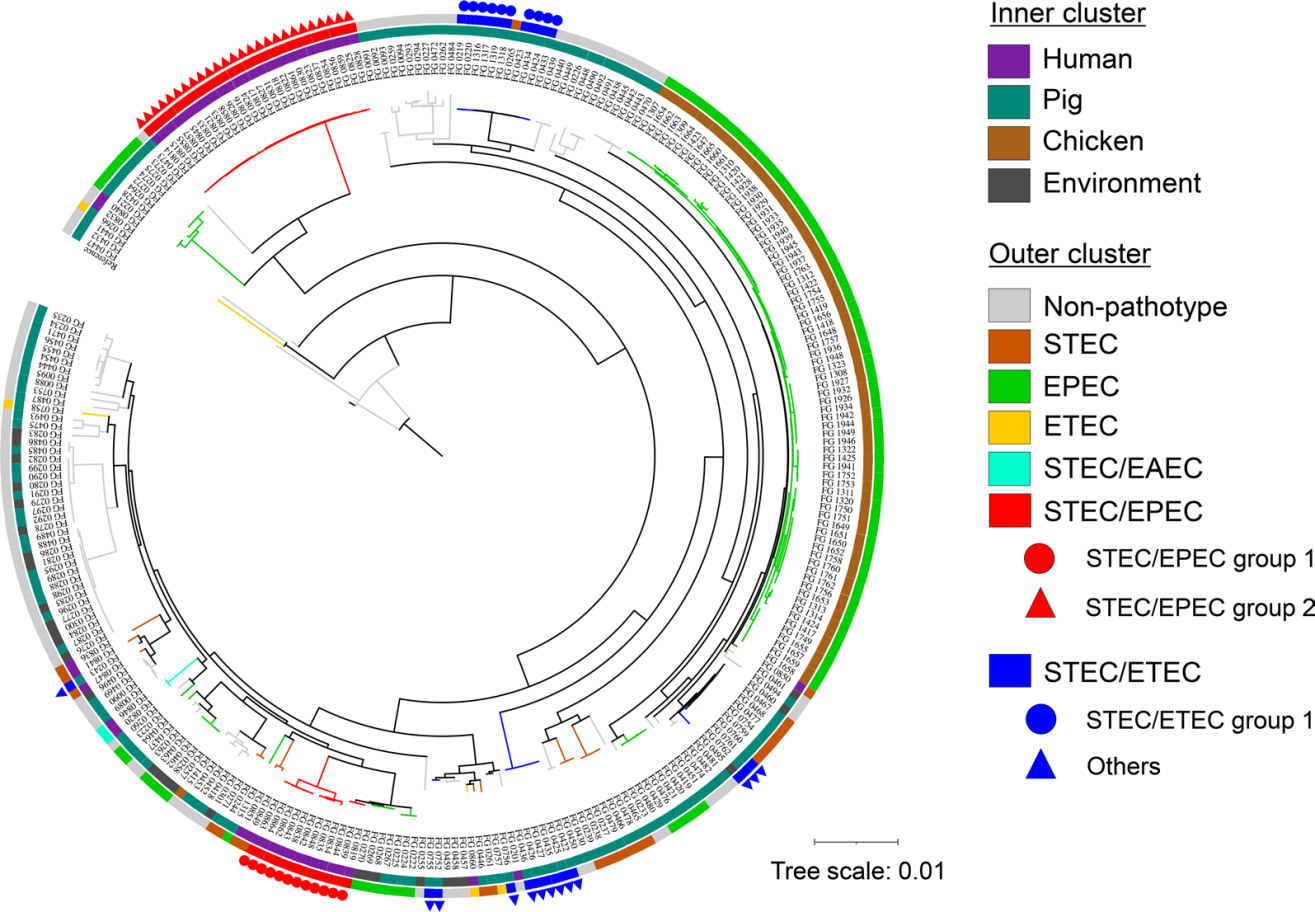
Phylogenetic relationships of 292 non-pathogenic and pathogenic *E. coli* isolates based on cgSNPs. The phylogenetic tree, which as inferred by method of a maximum-likelihood (ML), illustrates genetic relationships among *E. coli* isolates of mono-pathotypes (EAEC, EHEC, EPEC, ETEC, and STEC) and hybrid-pathotypes (STEC/EPEC, STEC/ETEC). Colored branches indicate different pathotypes as shown in the legend. For STEC/EPEC and STEC/ETEC hybrid-pathotypes, additional grouping was indicated. *Salmonella enterica* serovar Typhimurium strain LT2 was used as the outgroup for the phylogenetic analysis

Figure 1 shows the phylogenetic tree based on cgSNPs of 292 *E. coli* isolates used in this study. Overall, pathotypes did not form monophyletic groups, indicating that phylogenetic clustering does not correspond to pathotype. Although 76 EPEC mono-pathotype isolates clustered into a major group, the remaining 26 EPEC isolates were widely dispersed in the phylogenetic tree. Hybrid-pathotypes also did not form discrete clusters. In the core-genome phylogeny, two major groups (groups 1 and 2) were identified for STEC/EPEC hybrid-pathotype isolates. For STEC/ETEC hybrids, one major group (group 1) containing 10 isolates were observed, whereas the remining six isolates did not form into a single cluster (Fig. 1).

Notably, both STEC/EPEC groups were positioned closest to EPEC mono-pathotype isolates. Both groups of STEC/EPEC isolates from humans were clustered with EPEC mono-pathotype isolates from porcine sources. In contrast to the STEC/EPEC hybrid-pathotype isolates clustered with EPEC monotype isolates, the main STEC/ETEC hybrid group (blue circles) clustered nearest to non-pathogenic isolates, whereas some STEC/ETEC clusters (blue triangles) grouped with STEC mono-pathotype isolates (Fig. 1).

### Integration sites of LEE pathogenicity island and *stx* prophages

LEE pathogenicity islands (PAIs) were integrated into three chromosomal genes, *pheV*, *pheU*, and *selC*., in EPEC pathotype isolates. Those of the STEC/EPEC group 1 and its adjacent EPEC were integrated mainly into the *pheU* (11/19) and *pheV* (7/19). On the other hand, *selC* was the main integration site of LEE PAIs of STEC/EPEC group 2 and its adjacent EPEC isolates (22/33). It should be noted that the putative integration site was inferred from short-read-based draft assemblies, which may not accurately resolve the precise PAI–chromosome junction. Minimum spanning tree showed close relationships between LEE pathogenicity islands with similar structures, especially in those of STEC/EPEC group 2 and its near EPEC monotype (Supplementary Figure S1).

In the chromosomes of STEC pathotype isolates, *stx1* and *stx2* were integrated into *yehV* and *wrbA* loci, respectively.

### Synteny analysis of the LEE pathogenicity island

**Fig. 2:**
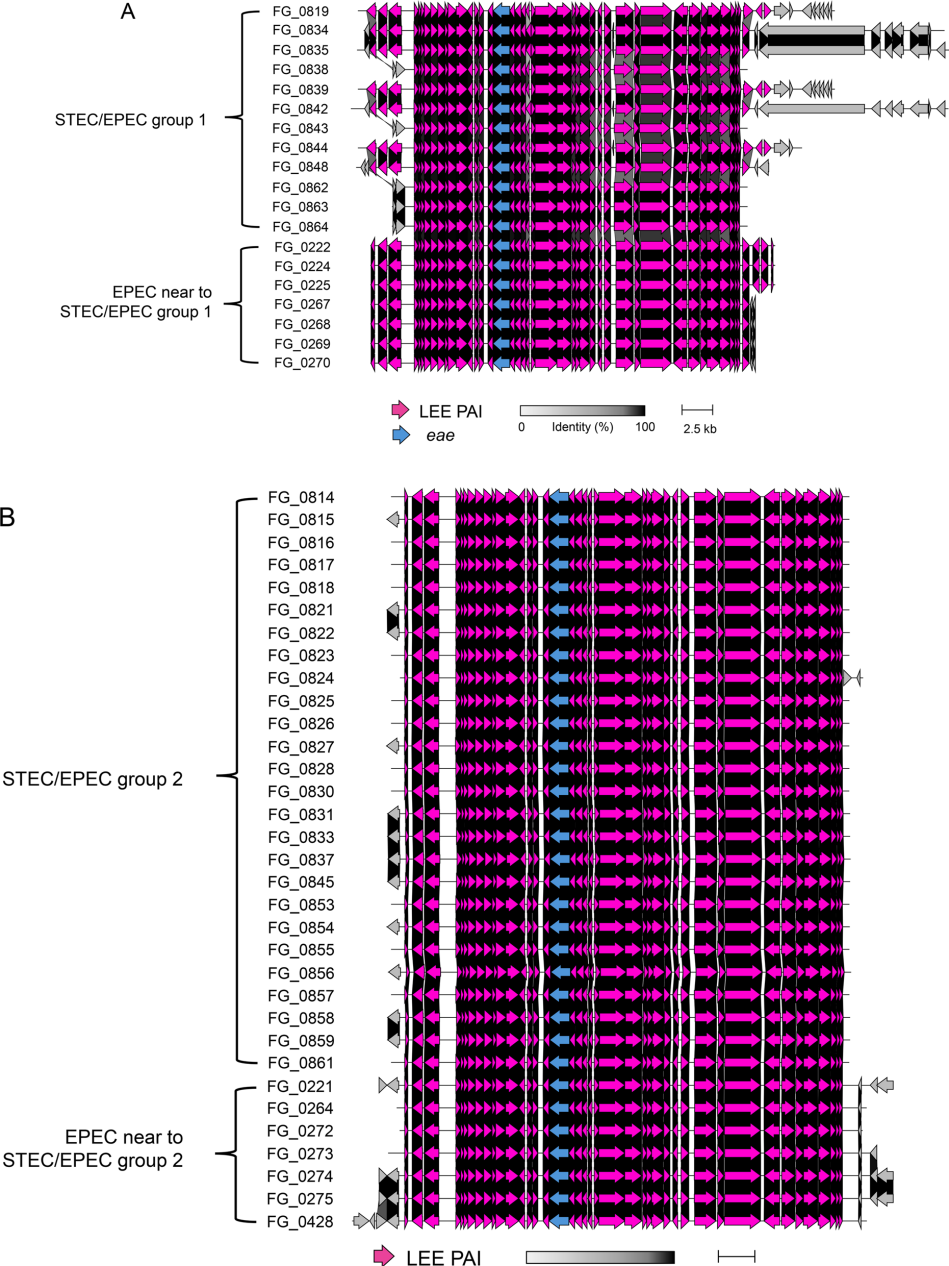

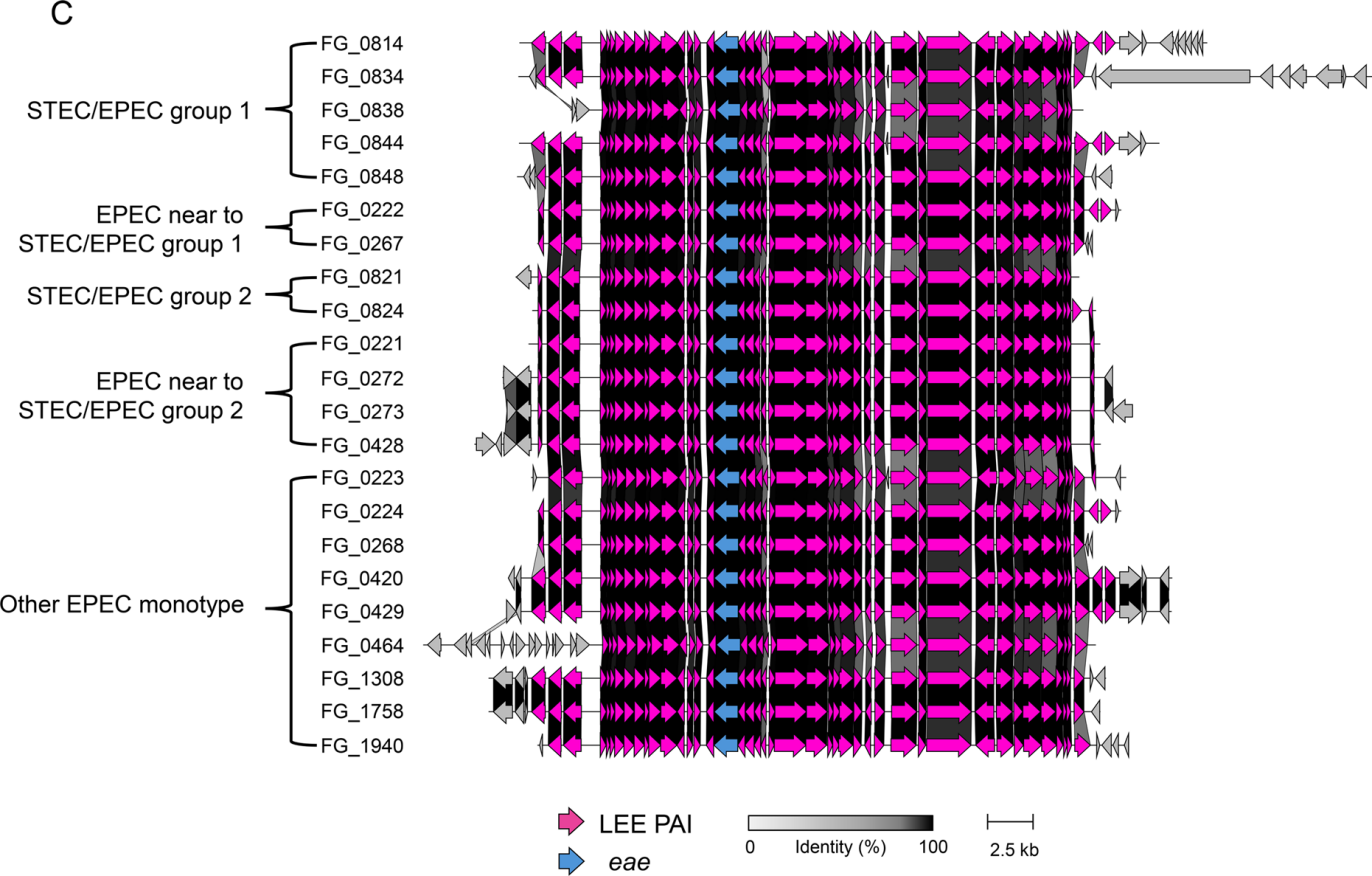
**S**ynteny analysis of the locus of enterocyte effacement pathogenicity island (LEE PAI) and its surrounding regions. **A** Comparison of the LEE PAI between STEC/EPEC group 1 and its phylogenetically related EPEC mono-pathotype isolates. **B** Comparison of the LEE PAI between STEC/EPEC group 2 and its neighboring EPEC mono-pathotype isolates. **C** Representative synteny of the LEE PAI across STEC/EPEC groups and randomly selected EPEC mono-pathotype isolates belonging to the major cluster containing isolates related to both hybrid groups. Pink arrows indicate genes within the LEE PAI. The gray-to-black scale bar represents the percentage of amino acid identity (0%–100%). The scale bar at the bottom denotes the genomic length (2.5 kb). The *eae* was highlighted in blue

To examine the structural conservation of the LEE PAI, a synteny analysis was performed on STEC/EPEC hybrid- and EPEC mono-pathotype isolates (Fig. 2). The LEE PAI displayed a highly conserved architecture, whereas gene order and orientation were largely collinear, with dense alignments across core LEE genes and only minor disruptions near island boundaries. STEC/EPEC group 1 showed a somewhat diverse structure in the flanking non-LEE PAI regions, some of which were similar to those of EPEC mono-pathotype isolates closely related to group 1 (Fig. 2A). In contrast, the homogeneous structure of the flanking non-LEE PAI regions of STEC/EPEC group 2 differed from that of STEC/EPEC group 1, but was very similar to that of its closely related EPEC mono-pathotype isolates (Fig. 2B). EPEC mono-pathotype isolates that did not cluster with hybrids showed distinct LEE structural variation (Fig. 2C).

In addition, STEC/EPEC hybrid and EPEC monotype isolates harbored the same alpha-intimin subtype, *eaeA*, which encoded a typical EPEC intimin adherence protein. BLAST analysis showed that *tir* encoding translocated intimin receptor exhibited the nucleotide identity with 97.5% to 100% among STEC/EPEC hybrid and EPEC monotype isolates.

### Accessory gene repertoire

**Fig. 3:**
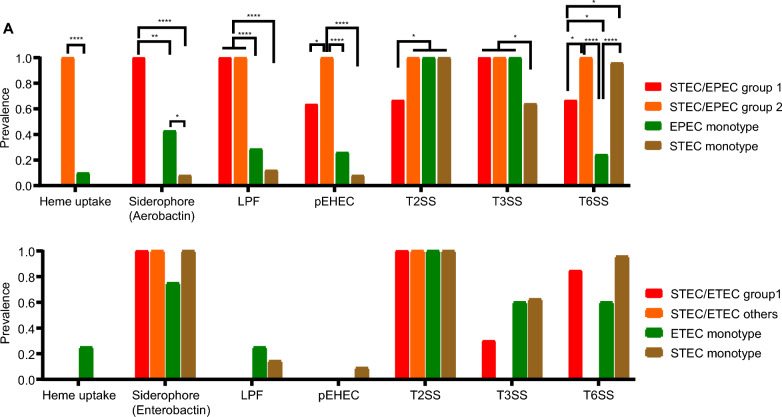
Prevalence of accessory gene repertoire associated with heme uptake, siderophore, LPF, pEHEC, T2SS, T3SS, and T6SS, in two groups of STEC/EPEC hybrid-pathotype and EPEC and STEC mono-pathotypes (**A**) and in STEC/ETEC hybrid-pathotype and ETEC and STEC mono-pathotypes (**B**). Statistical significance is indicated as follows: *p < 0.05; **p < 0.01; ***p < 0.001; ****p < 0.0001

We examined an expanded accessory gene repertoire which is hypothesized to reflect enhanced bacterial fitness (Fig. 3). For the STEC/EPEC hybrids, we built a combined pangenome using Panaroo (v1.2.5) with its default parameters, from the STEC/EPEC subgroups together with the EPEC and STEC mono-pathotype sets. The annotated genomes were processed using a sequence identity threshold of 95%. The genes were partitioned genes into core and accessory sets, and removed core genes which was defined as those present in ≥ 99% of the genomes. The same workflow was applied for STEC/ETEC hybrids. Group-specific enrichment was assessed using Fisher’s exact tests with Benjamini–Hochberg correction.

Genes associated with heme uptake were significantly enriched in STEC/EPEC group 2, suggesting an alternative iron-acquisition strategy (Fig. 3A). No heme-uptake genes were identified in group 1. Conversely, STEC/EPEC group 1 possessed numerous siderophore-associated loci, none of which were found in group 2. These siderophore genes were also enriched relative to mono-pathotype isolates. Long polar fimbriae (LPF) and pEHEC-like plasmid components were highly represented in both STEC/EPEC groups, indicating conserved adherence and EHEC-linked plasmid features.

T2SS-associated genes were fewer in STEC/EPEC group 1 than the other groups. T3SS-associated genes were present consistently in STEC/EPEC hybrid isolates and EPEC mono-pathotype isolates, but absent in STEC mono-pathotype isolates. For T6SS, STEC/EPEC group 2 and STEC mono-pathotype isolates contained more genes that the other two groups. On the other hand, the STEC/ETEC hybrid strains did not harbor significantly enriched genes compared to the mono-pathotypes.

### Virulence assay

**Fig. 4:**
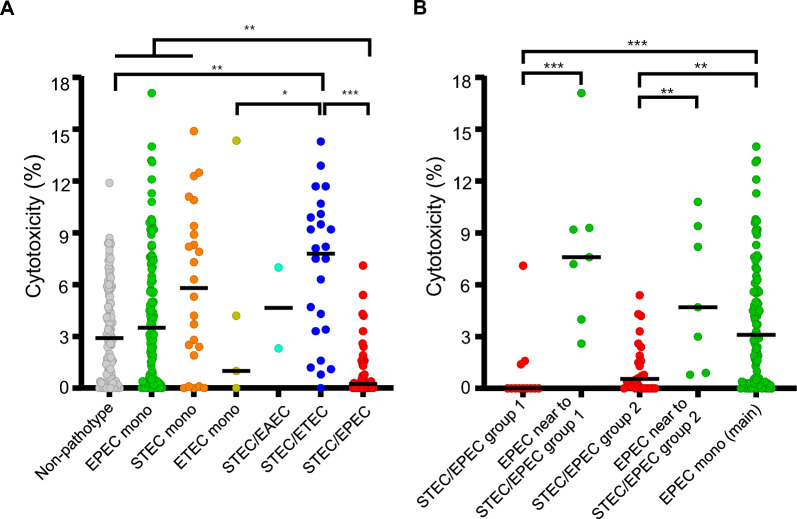
Lactate dehydrogenase (LDH) assay results, representing cytotoxic effect on different pathotypes of *E. coli* strains. **A** Cytotoxicity levels across different pathotypes, including mono and hybrid pathotypes. **B** A detailed comparison of STEC/EPEC groups (groups 1 and 2) with their phylogenetically neighboring EPEC mono-pathotype isolates and the main EPEC mono-pathotype cluster. Statistical significance is indicated as follows: *p < 0.05; **p < 0.01; ***p < 0.001

Cytotoxicity of *E. coli* isolates was measured using an LDH assay. The results showed broad variability among both non-pathogenic and pathogenic isolates (Fig. 4). When comparing hybrid pathotypes with their mono-pathotype counterparts, the two hybrid types showed opposite trends. STEC/ETEC hybrids exhibited significantly higher cytotoxicity compared to ETEC mono-pathotypes, and they showed a trend toward increased cytotoxicity compared to STEC isolates. In contrast, STEC/EPEC hybrids showed lower cytotoxicity than either EPEC or STEC mono-pathotypes.

Further analysis comparing the two STEC/EPEC groups with their corresponding EPEC mono-pathotype groups and unrelated mono-pathotype isolates showed that both hybrid groups had significantly lower cytotoxicity than their closely related and unrelated EPEC counterparts (Fig. 4B).

### Presence of virulence genes and cytotoxicity

**Fig. 5:**
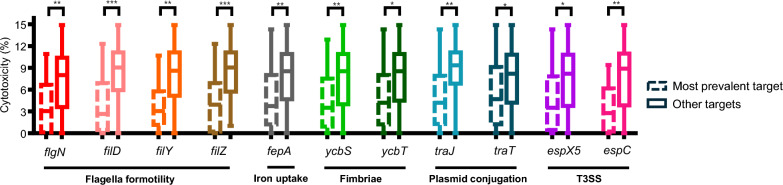
Genetic factors showing positive associations with increased cytotoxicity in LDH assay in STEC/ETEC hybrid-pathotype, which exhibited significantly higher cytotoxicity than STEC and ETEC mono-pathotypes. It was analyzed using SPLASH. Statistical significance is indicated as follows: *p < 0.05; ****p < 0.0001

We sought to identify genetic factors associated with increased virulence in specific hybrid groups using SPLASH. Based on metadata, analyses focused on the STEC/ETEC hybrid pathotype, which showed markedly elevated cytotoxicity compared with its constituent mono-pathotypes, STEC and ETEC. Across the 11 genes that remained significant after multiple-testing correction, the level of cytotoxicity was consistently higher in the ‘other-targets’ group than in the ‘most-prevalent-target’ group (Fig. 5). These genes belong to five functional modules, flagella motility, iron uptake, fimbriae, plasmid conjugation, and the type III secretion system (T3SS). This finding suggests that non-dominant target sequence types at these loci are associated with increased LDH release.

## Discussion

We performed whole genome sequencing analysis and investigated cytotoxicity based on the LDH assay for a large number of intestinal pathogenic *E. coli* isolates collected from humans, animals, and the environment in South Korea.

Since an outbreak in Germany in 2011, hybrid-pathotypes of pathogenic *E. coli* have been steadily reported [11]. STEC/EPEC hybrid-pathotype has been reported in several countries including South Korea [32–35]. Our study revealed a high frequency of the STEC/EPEC hybrid-pathotype. STEC/ETEC hybrid-pathotype isolates have also been identified worldwide [36–41]. STEC/EAEC hybrid strains were the causative agent of the German outbreak in 2011. Although they have been relatively infrequently detected thereafter [42], the two STEC/EAEC O104:H4 isolates identified in this study showed very high nucleotide identity with it. It is noted that not a few STEC isolates were identified as hybrids with ETEC, EPEC, or EAEC. In seventy percent of STEC isolates, additional virulence genes corresponding to different pathotypes were identified. This demonstrates the dynamic nature of the *E. coli* genome, emphasizing the need to pay attention to the virulence properties of hybrid-pathotypes.

In this study, each hybrid-pathotype was strongly associated with isolation source: STEC/EPEC from humans and STEC/ETEC from pigs. In addition, the hybrid-pathotypes are assumed to be formed on different genomic backbones. Notably, both groups of STEC/EPEC hybrid isolates showed close relationships with EPEC mono-pathotype isolates. However, MLST analysis indicated that STEC/EPEC group 2 may have different pathway, because ST11 was found in the STEC/EPEC hybrid isolates including O157:H7 and STEC mono-type isolates but not in monotypic EPEC isolates. Two groups of STEC/EPEC hybrid isolates might be emerged from different genomic background.

On the other hand, STEC/ETEC hybrid isolates were phylogenetically associated with STEC mono-pathotype isolates, although the relationships were less evident than those of STEC/EPEC hybrid isolates. A genome-based study showed that a particular genotype of STEC/EPEC hybrid might have emerged by the acquisition of STEC virulence genes by EPEC via phage [35]. Emergence of hybrid pathogenic *E. coli* causing serious disease by *stx*-converting phages has been reported in non-O157 strains from Germany [43]. Thus, we confirmed that several STEC/EPEC hybrid-pathotypes originated from EPEC strains. In addition, the emergence of STEC/EPEC hybrid-pathotype may not be a restricted event. EPEC isolates with H7, H11, or H2 antigens, which were closely associated with the STEC/EPEC hybrid isolates from humans, were collected from pigs. This indicates that STEC/EPEC hybrid-pathotypes in humans may originate from EPEC strains of pigs. It is postulated that the incorporation of STEC-associated factors, including *stx*, into EPEC strains might accelerate cross-infection between humans and pigs. The cross-infection between humans and animals and subsequent emergence of hybrid pathogenic strains further strengthens the One Health perspective.

*E. coli* O157:H7 variants have diverged through various evolutionary pathways [44]. The predominance of O157:H7, which has been globally recognized as a highly pathogenic serotype in *E. coli* [45, 46], among the STEC/EPEC hybrid-pathotype isolates should be noted. As far as we know, no hybrid-pathotypes have been previously recognized among O157:H7 isolates. These isolates showed a close relationship with other STEC/EPEC or EPEC O26:H11 isolates, which have been identified as important virulent pathogens among non-O157 *E. coli* [32, 47]. In this study, all EPEC isolates contained *eae* but *bfpA*, thus they are classified into aEPEC. As *bfpA* is usually harbored on plasmid, the loss of plasmid is assumed to be aEPEC in South Korea. The influence of loss of plasmid harboring *bfpA* was not evaluated because no tEPEC was not identified in the present study.

In contrast to STEC/EPEC hybrid-pathotype, STEC/ETEC hybrid-pathotypes might not emerge by the introduction of STEC factors into an ETEC backbone. Conversely, some groups of STEC/ETEC hybrid isolates showed close relationships with STEC mono-pathotype isolates. Among the STEC/ETEC hybrid isolates, only *stx2* gene was found unlike STEC/EPEC hybrid isolates. It may show that the origin of STEC/ETEC is different from STEC/EPEC. The hybrid-pathotypes may arise through diverse mechanisms.

In addition to STEC/EPEC and STEC/ETEC hybrid-pathotypes, two STEC/EAEC hybrid isolates were also identified, and they are supposed to have originated from EAEC isolates from pigs. They showed the same serotype, O104:H4, as the hybrid *E. coli* strains causing the German outbreak in 2011 [11]. Although it is currently unclear how the backbone differs according to the type of hybrid-pathotype and the epidemiological relevance of their isolation sources, further investigation into these topics seems necessary.

In this study, STEC/EPEC hybrid isolates showed low cytotoxicity based on the LDH assay. Lower cytotoxicity of both groups of STEC/EPEC hybrid isolates than their phylogenetically related EPEC mono-pathotype isolates indicated that the introduction of STEC factors into an EPEC backbone might be associated with reduced cytotoxicity. Impressively, our analyses indicate group-specific enrichment of accessory functions in STEC/EPEC hybrid-pathotypes. Siderophore-associated loci were universally present and significantly enriched in STEC/EPEC group 1, while heme-uptake genes were significantly enriched in group 2 after removing genes shared by STEC, EPEC, and STEC/EPEC. Siderophore and heme uptake are important factors contributing to bacterial pathogenicity [48–50], but the large accessory gene load in STEC/EPEC hybrids may impose a high fitness cost, prioritizing survival functions and reducing cytotoxic output. This hypothesis is consistent with a previous study showing that STEC/UPEC hybrid strains exhibited reduced survival under highly acidic conditions and decreased motility compared to canonical STEC and UPEC strains [51].

STEC/ETEC hybrid-pathotype isolates exhibited markedly higher cytotoxicity than their corresponding mono-pathotype isolates. In contrast to STEC/EPEC hybrids, STEC/ETEC hybrids showed no significant gene-content differences from mono-pathotypes beyond the canonical markers. Using SPLASH, we identified five gene modules associated with increased cytotoxicity in STEC/ETEC: pili involved in host-cell adhesion, plasmid conjugation, flagella, iron uptake (including siderophore-related genes), and the T3SS. These modules are well-established contributors to bacterial virulence [1, 7, 48–50, 52, 53].

Together, these findings suggest that STEC/ETEC hybrids may achieve enhanced pathogenicity through sequence-level variation in additional virulence-associated loci, potentially with a smaller fitness cost than large-scale gene acquisitions. However, we did not pinpoint the exact nucleotide changes at these loci using genome assemblies or read-mapping-based variant calling, nor did we perform expression analyses or functional experiments. Further work will therefore be required to validate the causal mechanisms and to rule out potential confounding effects.

The difference between STEC/EPEC and STEC/ETEC may be due to distinct paths leading to the formation of hybrid-pathotypes. Nonetheless, it is noteworthy that changes in cytotoxicity might be linked to the incorporation of additional genomic regions. The cytotoxicity measured by LDH may not represent the entire spectrum of virulence in humans or other animals, and thus other assays, for example, sorbitol fermenting (SF) and β-glucuronidase (GUD) assays, may reveal more definitive results on the virulence of hybrid-pathotypes with more virulence factors.

## Conclusions

In the present study, we compared whole genome sequences and cytotoxicity of intestinal pathogenic *E. coli* isolates. We found many hybrid-pathotype isolates. We identified substantial genomic complexity in hybrid-pathotypes, reflecting repeated independent emergence of hybrid-pathotypes, in addition to the close association of hybrid type with isolation source. While some STEC/EPEC might originate by incorporation of STEC factors into an EPEC genome backbone, most STEC/ETEC hybrid isolates might have formed from an STEC backbone. In addition to the genomic complexity inherent in hybrid-pathotypes, they exhibited divergent virulence patterns. STEC/EPEC hybrids showed reduced cytotoxicity, whereas STEC/ETEC hybrids demonstrated increased cytotoxicity compared to mono-pathotypes. Particularly, the emergence of STEC/EPEC and STEC/ETEC hybrid-pathotypes with highly pathogenic serotypes such as O157:H7 and O100:H30, respectively, may pose an increased risk to public health. This underscores the necessity for comprehensive surveillance and prompt identification strategies to mitigate the potential risk of outbreaks associated with these emerging hybrid isolates.

## Supplementary Information

Supplementary Material 1.

Supplementary Material 2.

Supplementary Material 3.

## Data Availability

All materials are available upon request to the corresponding author.
