# Supplementary material for: Comparison of hybrid-and mono-pathotype Escherichia coli isolates from South Korea based on whole genome analysis and cytotoxicity assay
Source: J Biomed Sci. 2026 Apr 13;33:40. doi: 10.1186/s12929-026-01243-0 (PMC13072654; doi:10.1186/s12929-026-01243-0)
Supplement: Supplementary file 1 — Supplementary Material 1. [file 12929_2026_1243_MOESM1_ESM.pptx]

## Slide 1
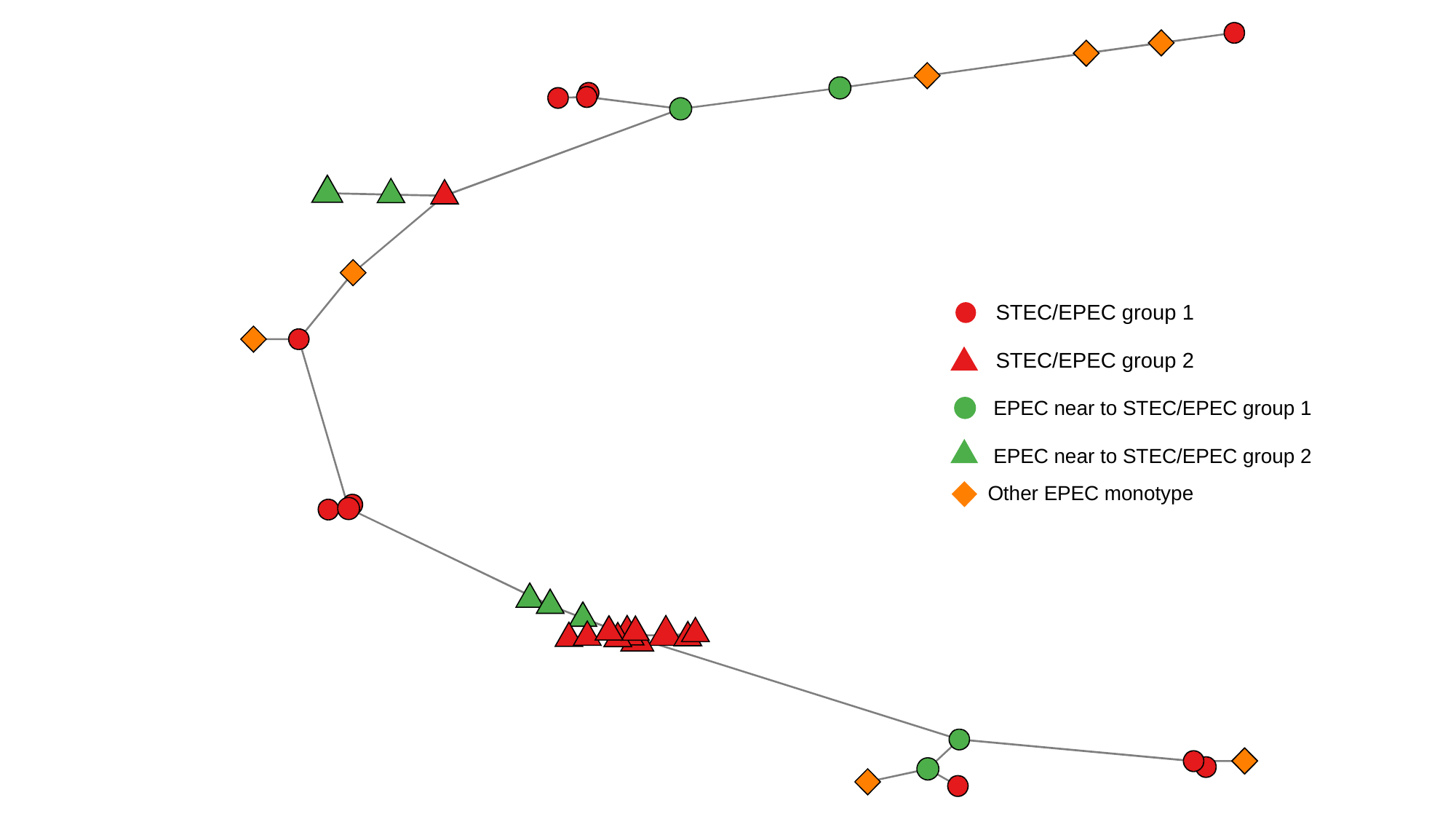

STEC/EPEC group 1
STEC/EPEC group 2
EPEC near to STEC/EPEC group 1
EPEC near to STEC/EPEC group 2
Other EPEC monotype
