# Supplementary material for: Comparison of hybrid-and mono-pathotype Escherichia coli isolates from South Korea based on whole genome analysis and cytotoxicity assay
Source: J Biomed Sci. 2026 Apr 13;33:40. doi: 10.1186/s12929-026-01243-0 (PMC13072654; doi:10.1186/s12929-026-01243-0)
Supplement: Supplementary file 3 — Supplementary Material 3. [file 12929_2026_1243_MOESM3_ESM.pdf]

Supplementary Table S1. Distribution of EPEC pathotypes across different O-antigens and H-antigens.

	-	H2	H7	H11	H15	H16	H18	H21	H25	H28	H30	H34	H40	H42	H49	H52	Total
-	5							32		1	4		9			1	52
O7								1									1
O17							1					1					2
O23					1												1
O26				5													5
O49													1				1
O51														2	3		5
O71													1				1
O76			1														1
O78								2									2
O88									1								1
O100											2						2
O103		4															4
O108						1											1
O123/O186													3				3
O132														8			8
O145	2																2
O153/O178			2														2
O177										6							6
O178			2														2
Total	7	4	5	5	1	1	1	35	1	7	6	1	16	8	3	1	102

Supplementary Table S2. Distribution of STEC pathotypes across different O-antigens and H-antigens.

	H4	H7	H8	H9	H10	H14	H19	H30	H38	Total
-									4	4
O8	1			1			1			3
O9				5				3		8
O91						2				2
O100								1		1
O111			1							1
O121					2					2
O157		3								3
Total	1	3	1	6	2	2	1	4	4	24

Supplementary Table S3. Distribution of ETEC pathotypes across different O-antigens and H-antigens.

	H2	H8	H9	H18	Total
O7				1	1
O9			1		1
O111		1			1
O174	1				1
Total	1	1	1	1	4

Supplementary Table S4. Distribution of non-pathotype *E. coli* across different O-antigens and H-antigens.

	H2	H4	H5	H7	H8	H9	H10	H11	H12	H14	H16	H18	H21	H23	H27	H28	H30	H32	H34	H38	H40	H45	H49	H51	Total
-				1		1							1	9	2	1		4					1		20
O6																							1		1
O8	1			2		6																	1		10
O9						2																			3
O13/O135		1															2								3
O15								1														3			4
O18				3														1							4
O26								1																	1
O33												1													1
O39																		2							2
O45																						2			2
O54													1												1
O59			1																						1
O62																	5								5
O70							1																		1
O71			1												1										2
O84										1						2									3
O88					1			1																	2
O91																13									13
O99																				3					3
O109																						3			3
O130												1													1
O136																					1				1
O149									1																1
O153							1																		1
O157				1																					1
O160											1								2						3
O162							1																		1

O171																						1			1
O174		2																							2
O179			1																						1
O180																								1	1
Total	1	3	3	7	1	9	3	3	1	1	1	2	2	11	3	16	8	5	2	3	1	9	3	1	99

Supplementary Table S5. Distribution of STEC/EPEC pathotypes across different O-antigens and H-antigens.

	H2	H4	H7	H8	H10	H11	H21	H25	H42	H43	Total
-							1				1
O4								1			1
O21											1
O26						1					1
O69						1					1
O103	3										3
O104		1									1
O111				3							3
O115					1						1
O132									1		1
O157			22							1	23
O179				1							1
Total	3	1	22	4	1	2	1	2	1	1	38

Supplementary Table S6. Distribution of STEC/ETEC pathotypes across different O-antigens and H-antigens.

	H9	H16	H21	H30	H40	Total
-	1		1	1		3
O8	3					3
O9	2					2
O100				8		8
O123/O186					1	1
O159		6				6
Total	6	6	1	9	1	23
